# Supplementary material for: Structurally Different Exogenic Brassinosteroids Protect Plants under Polymetallic Pollution via Structure-Specific Changes in Metabolism and Balance of Cell-Protective Components
Source: Molecules. 2023 Feb 22;28(5):2077. doi: 10.3390/molecules28052077 (PMC10003821; doi:10.3390/molecules28052077)
Supplement: Supplementary file 1 [file molecules-28-02077-s001.zip › molecules-2193816_S1.pdf]

Table S1. The effects of heavy metal stress and treatment with brassinosteroids on the growth parameters of barley plants.

		Stem length, cm	Root length, cm	Leaf area, cm^2	Fresh weight, g
	**Control**	12.78 ± 0.18	17.39 ± 0.16	13.84 ± 0.19	1.15 ± 0.02
	**Stress**	10.92 ± 0.18*	10.81 ± 0.23*	10.94 ± 0.23*	0.86 ± 0.02*
**1-day pretreatment + stress**	**0.1 nM HBL**	10.46 ± 0.30	11.02 ± 0.26	10.66 ± 0.33	0.85 ± 0.03
	**10 nM HBL**	11.32 ± 0.24	10.58 ± 0.23	11.72 ± 0.32	0.92 ± 0.02
	**0.1 nM HCS**	12.14 ± 0.15[a]	12.01 ± 0.11[a]	11.49 ± 0.13	0.90 ± 0.02
	**10 nM HCS**	12.52 ± 0.11[a]	12.35 ± 0.15[a]	12.32 ± 0.13[a]	0.97 ± 0.02[a]
**10-days treatment + stress**	**0.1 nM HBL**	11.18 ± 0.19	11.95 ± 0.21[a]	12.13 ± 0.29[a]	0.92 ± 0.03
	**10 nM HBL**	11.76 ± 0.22[a]	10.64 ± 0.22	12.02 ± 0.29[a]	0.89 ± 0.03
	**0.1 nM HCS**	12.91 ± 0.20[a]	13.04 ± 0.14[a]	11.35 ± 0.12[a]	0.92 ± 0.02
	**10 nM HCS**	12.48 ± 0.13[a]	12.17 ± 0.14[a]	12.46 ± 0.12[a]	0.94 ± 0.01[a]

Mean values ± SE are given. Pairwise comparisons of the means with controls at corresponding time points were performed using Student's t-test. Significant differences at $p < 0.05$ from the control are denoted by asterisk (*), and significant differences between “Stress” and Stress with HBL or with HCS variants are denoted by (a).
