# Supplementary material for: Structurally Different Exogenic Brassinosteroids Protect Plants under Polymetallic Pollution via Structure-Specific Changes in Metabolism and Balance of Cell-Protective Components
Source: Molecules. 2023 Feb 22;28(5):2077. doi: 10.3390/molecules28052077 (PMC10003821; doi:10.3390/molecules28052077)
Supplement: Supplementary file 1 [file molecules-28-02077-s001.zip › molecules-2193816_S2.pdf]

Table S2 The effects of heavy metal stress and treatment with brassinosteroids on the primary photosynthetic processes in barley plants.

	Y (II)	ETR	qP	NPQ	F_v/F_m
**Control**	0.68 ± 0.01	25.08 ± 0.32	0.77 ± 0.02	0.61 ± 0.02	0.85 ± 0.00
**Stress**	0.71 ± 0.01	26.02 ± 0.32	0.81 ± 0.02	0.60 ± 0.02	0.83 ± 0.00
**10 nM HBL+stress**	0.66 ± 0.02	24.64 ± 0.74	0.77 ± 0.02	0.58 ± 0.04	0.83 ± 0.01
**10 nM HCS +stress**	0.74 ± 0.02	26.96 ± 0.60	0.78 ± 0.03	0.62 ± 0.02	0.84 ± 0.01
