# Supplementary material for: Structurally Different Exogenic Brassinosteroids Protect Plants under Polymetallic Pollution via Structure-Specific Changes in Metabolism and Balance of Cell-Protective Components
Source: Molecules. 2023 Feb 22;28(5):2077. doi: 10.3390/molecules28052077 (PMC10003821; doi:10.3390/molecules28052077)
Supplement: Supplementary file 1 [file molecules-28-02077-s001.zip › molecules-2193816_S3.pdf]

Table S3 The effects of heavy metal stress and treatment with brassinosteroids on concentrations of photosynthetic pigments (mg/g fresh weight) in barley plants.

	**Chl *a***	**Chl *b***	**Car**
**Control**	2.01 ± 0.10	0.50 ± 0.02	0.53 ± 0.03
**Stress**	0.61 ± 0.07*	0.16 ± 0.02*	0.22 ± 0.03*
**10 nM HBL+stress**	0.70 ± 0.03	0.18 ± 0.01	0.23 ± 0.01
**10 nM HCS +stress**	1.05 ± 0.09[a]	0.23 ± 0.03	0.34 ± 0.02[a]

Mean values ± SE are given. Pairwise comparisons of the means with controls at corresponding time points were performed using Student's t-test. Significant differences at $p < 0.05$ from the control are denoted by asterisk (*), and significant differences between "Stress" and Stress with HBL or with HCS variants are denoted by (a).
