# Supplementary material for: Structurally Different Exogenic Brassinosteroids Protect Plants under Polymetallic Pollution via Structure-Specific Changes in Metabolism and Balance of Cell-Protective Components
Source: Molecules. 2023 Feb 22;28(5):2077. doi: 10.3390/molecules28052077 (PMC10003821; doi:10.3390/molecules28052077)
Supplement: Supplementary file 1 [file molecules-28-02077-s001.zip › molecules-2193816_S4.pdf]

Table S4. The effects of heavy metal stress and treatment with brassinosteroids on the content of TBARS and activity of antioxidant enzymes – SOD and POD in barley plants.

	**TBARS, ng/g fresh weight**		**SOD, U/g protein**		**POD, U/g protein in min**	
	Root	Shoot	Root	Shoot	Root	Shoot
**Control**	18.95 ± 1.10	93.14 ± 3.00	8.78 ± 0.22	42.24 ± 5.65	272.32 ± 63.22	24.60 ± 3.63
**Stress**	44.99 ± 2.77	89.01 ± 4.25	9.45 ± 6.98	33.51 ± 3.42	642.20 ± 114.19*	27.38 ± 4.78
**10 nM HBL +stress**	32.36 ± 2.48[a]	123.02 ± 6.10 [a]	9.54 ± 2.68	42.42 ± 6.98	1620.40 ± 133.25[a]	62.15 ± 6.01[a]
**10 nM HCS +stress**	60.18 ± 3.01[a]	66.26 ± 4.10	9.39 ± 2.13	44.24 ± 4.87	688.52 ± 77.93	25.66 ± 3.03

Mean values ± SE are given. Pairwise comparisons of the means with controls at corresponding time points were performed using Student's t-test. Significant differences at $p < 0.05$ from the control are denoted by asterisk (*), and significant differences between "Stress" and Stress with HBL or with HCS variants are denoted by (a).
