# Supplementary material for: Structurally Different Exogenic Brassinosteroids Protect Plants under Polymetallic Pollution via Structure-Specific Changes in Metabolism and Balance of Cell-Protective Components
Source: Molecules. 2023 Feb 22;28(5):2077. doi: 10.3390/molecules28052077 (PMC10003821; doi:10.3390/molecules28052077)
Supplement: Supplementary file 1 [file molecules-28-02077-s001.zip › molecules-2193816_S5.pdf]

Table S5. The effects of heavy metal stress and treatment with brassinosteroids on the elemental content (μg/g dry weight) and translocation factors in roots of barley plants.

	Na	Al	K	Mn	Cu	Mg	P	Ca	Zn	Pb	Cd	Ni
**Control**	11,862±1,778	114±15	5150±327	62±4	15±0.8	1338±38	1,356±48	1232±127	40±3	0.62±0.06	1.20±0.1	0.82±0.06
**Stress**	13,410±290	1210±140*	8208±353*	997±92*	323±25*	996±61*	2121±50*	1985±235*	768±37*	24.5±1.8*	204±17*	478±33*
**10 nM HBL+stress**	17,771±421[a]	919±17	4039±266[a]	668±67[a]	120±14[a]	1594±51[a]	2239±54	1576±77	416±28[a]	4.63±0.33[a]	173±17	258±21[a]
**10 nM HCS +stress**	18,006±54[a]	468±37[a]	4908±284[a]	600±45[a]	136±7[a]	1555±48 [a]	2011±161	1781±121	366±25[a]	7.79±0.36[a]	152±13	259±25[a]
