# Supplementary material for: Structurally Different Exogenic Brassinosteroids Protect Plants under Polymetallic Pollution via Structure-Specific Changes in Metabolism and Balance of Cell-Protective Components
Source: Molecules. 2023 Feb 22;28(5):2077. doi: 10.3390/molecules28052077 (PMC10003821; doi:10.3390/molecules28052077)
Supplement: Supplementary file 1 [file molecules-28-02077-s001.zip › molecules-2193816_S6.pdf]

Table S6. The effects of heavy metal stress and treatment with brassinosteroids on the elemental content (µg/g dry weight) and translocation factors in shoots of barley plants.

	Na	Al	K	Mn	Cu	Mg	P	Ca	Zn	Pb	Cd	Ni
**Control**	3105±106	241±51	15,218±612	27±2	9±0.5	2410±45	1646±104	8060±391	28±4	0.12±0.02	0,09±0.003	0.66±0.05
**Stress**	1829±79*	180±14	17,862±136*	255±41*	14±0.9*	1491±59*	1569±46	3632±318*	133±10*	0.36±0.04*	23±2*	29.8±2.8*
**10 nM HBL+stress**	2264±89[a]	568±59[a]	19,869±219[a]	202±14	11±0.9	1876±125[a]	1767±40	3192±318	87±2[a]	0.95±0.06[a]	14±1[a]	23.6±1
**10 nM HCS +stress**	2638±127[a]	309±59[a]	15,524±986[a]	119±10	12±0.8	1850±31[a]	1342±52	4123±327	79±3[a]	0.41±0.03[a]	13±1[a]	15.2±0.4[a]

Mean values ± SE are given. Pairwise comparisons of the means with controls at corresponding time points were performed using Student's t-test. Significant differences at $p < 0.05$ from the control are denoted by asterisk (*), and significant differences between "Stress" and Stress with HBL or with HCS variants are denoted by (a).
