# Supplementary material for: Structurally Different Exogenic Brassinosteroids Protect Plants under Polymetallic Pollution via Structure-Specific Changes in Metabolism and Balance of Cell-Protective Components
Source: Molecules. 2023 Feb 22;28(5):2077. doi: 10.3390/molecules28052077 (PMC10003821; doi:10.3390/molecules28052077)
Supplement: Supplementary file 1 [file molecules-28-02077-s001.zip › molecules-2193816_S7.pdf]

Table S7. The effects of heavy metal stress and treatment with brassinosteroids on the expression of genes involved in metal detoxification in barley plants.

	HMA1		*HMA4*		*ALMT*		*MTP*		*PAA1*	
	Root	Shoot	Root	Shoot	Root	Shoot	Root	Shoot	Root	Shoot
**Control**	1.00 ± 0.04	1.00 ± 0.05	1.00 ± 0.07	1.00 ± 0.06	1.00 ± 0.00	1.00 ± 0.00	1.00 ± 0.04	1.00 ± 0.04	1.00 ± 0.08	1.00 ± 0.06
**Stress**	1.15 ± 0.05	1.58 ± 0.03*	0.90 ± 0.03	0.78 ± 0.04*	4.73 ± 0.07*	0.45 ± 0.02*	1.01 ± 0.04	0.72 ± 0.05*	0.78 ± 0.06	0.94 ± 0.04
**10 nM HBL+stress**	1.17 ± 0.05	2.99 ± 0.05 [a]	1.00 ± 0.09	1.24 ± 0.08 [a]	4.66 ± 0.1	2.25 ± 0.03 [a]	0.74 ± 0.06 [a]	0.96 ± 0.05	0.94 ± 0.1	1.46 ± 0.04
**10 nM HCS +stress**	0.89 ± 0.04	1.52 ± 0.05	0.78 ± 0.03	0.67 ± 0.03	1.3 ± 0.1 [a]	0.83 ± 0.03 [a]	0.47 ± 0.02 [a]	0.48 ± 0.04 [a]	0.70 ± 0.05	0.88 ± 0.05

Mean values ± SE are given. Pairwise comparisons of the means with controls at corresponding time points were performed using Student's t-test. Significant differences at $p < 0.05$ from the control are denoted by asterisk (*), and significant differences between “Stress” and Stress with HBL or with HCS variants are denoted by (a).
