# Supplementary material for: Structurally Different Exogenic Brassinosteroids Protect Plants under Polymetallic Pollution via Structure-Specific Changes in Metabolism and Balance of Cell-Protective Components
Source: Molecules. 2023 Feb 22;28(5):2077. doi: 10.3390/molecules28052077 (PMC10003821; doi:10.3390/molecules28052077)
Supplement: Supplementary file 1 [file molecules-28-02077-s001.zip › molecules-2193816_S8.pdf]

Table S8. The effects of heavy metal stress on brassinosteroids content (ng/g dry weight) in roots and shoots of barley plants.

	**β-lactone**		**6-ketone**	
	Root	Shoot	Root	Shoot
**Control**	3.07 ± 0.29	5.20 ± 0.80	2.43 ± 0.48	7.10 ± 0.61
**Stress**	3.89 ± 0.30	4.68 ± 0.46	2.35 ± 0.53	13.50 ± 2.40*

Mean values ± SE are given. Pairwise comparisons of the means with controls at corresponding time points were performed using Student's t-test. Significant differences at $p < 0.05$ from the control are denoted by asterisk (*), and significant differences between "Stress".
